# Effort–Reward Imbalance at Work and Incident Coronary Heart Disease: A Multicohort Study of 90,164 Individuals

**DOI:** 10.1097/EDE.0000000000000666

**Published:** 2017-06-01

**Authors:** Nico Dragano, Johannes Siegrist, Solja T. Nyberg, Thorsten Lunau, Eleonor I. Fransson, Lars Alfredsson, Jakob B. Bjorner, Marianne Borritz, Hermann Burr, Raimund Erbel, Göran Fahlén, Marcel Goldberg, Mark Hamer, Katriina Heikkilä, Karl-Heinz Jöckel, Anders Knutsson, Ida E. H. Madsen, Martin L. Nielsen, Maria Nordin, Tuula Oksanen, Jan H. Pejtersen, Jaana Pentti, Reiner Rugulies, Paula Salo, Jürgen Schupp, Archana Singh-Manoux, Andrew Steptoe, Töres Theorell, Jussi Vahtera, Peter J. M. Westerholm, Hugo Westerlund, Marianna Virtanen, Marie Zins, G. David Batty, Mika Kivimäki

**Affiliations:** From the a Institute of Medical Sociology, Medical Faculty, University of Düsseldorf, Düsseldorf, Germany; b Clinicum, Faculty of Medicine, University of Helsinki, Helsinki, Finland; c Institute of Environmental Medicine, Karolinska Institutet, Stockholm, Sweden; d School of Health Sciences, Jönköping University, Jönköping, Sweden; e Stress Research Institute, Stockholm University, Stockholm, Sweden; f Centre for Occupational and Environmental Medicine, Stockholm County Council, Sweden; g National Research Centre for the Working Environment, Copenhagen, Denmark; h Unit of Social Medicine, Frederiksberg University Hospital, Copenhagen, Denmark; i Federal Institute for Occupational Safety and Health (BAuA), Berlin, Germany; j Institute for Medical Informatics, Biometry, and Epidemiology, Faculty of Medicine, University Duisburg-Essen, Essen, Germany; k The National Agency for Special Needs Education and Schools, Härnösand, Sweden; l Paris Descartes University, Paris, France; m Inserm U1018, University Paris Saclay, France; n Department of Epidemiology and Public Health, University College London, London, United Kingdom; o School of Sport, Exercise and Health Sciences, University of Loughborough, Loughborough, United Kingdom; p Department of Health Services Research and Policy, London School of Hygiene and Tropical Medicine, London, United Kingdom; q Clinical Effectiveness Unit, The Royal College of Surgeons, London, United Kingdom; r Department of Health Sciences, Mid Sweden University, Sundsvall, Sweden; s AS3 Employment, AS3 Companies, Viby J, Denmark; t Department of Psychology, Umeå University, Umeå, Sweden; u Finnish Institute of Occupational Health, Helsinki, Tampere and Turku, Finland; v The Danish National Centre for Social Research, Copenhagen, Denmark; w Department of Public Health and Department of Psychology, University of Copenhagen, Copenhagen, Denmark; x Department of Psychology, University of Turku, Turku, Finland; y German Institute for Economic Research, Berlin, Germany; z Department of Public Health, University of Turku, Turku, Finland; aa Turku University Hospital, Turku, Finland; bb Occupational and Environmental Medicine, Uppsala University, Uppsala, Sweden.

## Abstract

**Background:**

Epidemiologic evidence for work stress as a risk factor for coronary heart disease is mostly based on a single measure of stressful work known as job strain, a combination of high demands and low job control. We examined whether a complementary stress measure that assesses an imbalance between efforts spent at work and rewards received predicted coronary heart disease.

**Methods:**

This multicohort study (the “IPD-Work” consortium) was based on harmonized individual-level data from 11 European prospective cohort studies. Stressful work in 90,164 men and women without coronary heart disease at baseline was assessed by validated effort–reward imbalance and job strain questionnaires. We defined incident coronary heart disease as the first nonfatal myocardial infarction or coronary death. Study-specific estimates were pooled by random effects meta-analysis.

**Results:**

At baseline, 31.7% of study members reported effort–reward imbalance at work and 15.9% reported job strain. During a mean follow-up of 9.8 years, 1,078 coronary events were recorded. After adjustment for potential confounders, a hazard ratio of 1.16 (95% confidence interval, 1.00–1.35) was observed for effort–reward imbalance compared with no imbalance. The hazard ratio was 1.16 (1.01–1.34) for having either effort–reward imbalance or job strain and 1.41 (1.12–1.76) for having both these stressors compared to having neither effort–reward imbalance nor job strain.

**Conclusions:**

Individuals with effort–reward imbalance at work have an increased risk of coronary heart disease, and this appears to be independent of job strain experienced. These findings support expanding focus beyond just job strain in future research on work stress.

## Abstract 2

Supplemental Digital Content is available in the text.

Stressful working conditions are common in the lives of workers in modern economies.^1^ This is a challenge for prevention as chronic or high-dosage stress may adversely affect cardiovascular health.^2,3^ Cohort studies from different countries have found an association between stressful working conditions and increased risk of subsequent coronary heart disease events among employees.^2,4–7^ In the majority of these studies, work stress was conceptualized and measured according to the “job strain” (also known as demand–control) model.^8^ This conception posits that stress results from exposure to a job characterized by high psychological demands in combination with a low degree of task control or decision latitude.^9,10^ In times of economic globalization, growing rationalization, work intensification, job insecurity, and income inequality have become commonplace, and additional aspects of work might be important for inducing stress in employees. Some of these stress properties comprise the “effort–reward imbalance” model, a complementary model of stressful work.^11^

According to the effort–reward imbalance model, stress is generated by the recurrent experience of a failed reciprocity between the effort spent at work (e.g., pace of work, workload, time spend at work) and the rewards received in turn (“high cost–low gain” condition). In addition to wage and salary, rewards include nonmaterial aspects, such as esteem, recognition, promotion prospects, and job security. In four individual prospective studies from Germany, Great Britain, and Finland, employees with an imbalance between high effort and low reward had an increased risk of incident coronary heart disease or cardiovascular mortality.^12–15^ In one of these studies, effect estimates were additionally adjusted for job strain and as the association was not substantially attenuated, there is a suggestion that the effort–reward imbalance model may have its own predictive utility for coronary heart disease risk.^12^ However, owing to the small number of observations and independent studies, it is unknown whether these results are generalizable to different settings and across socioeconomic groups.^5,7,16^ Furthermore, the available evidence is limited because of a restricted range of occupations, crude measurements of effort–reward imbalance in some studies, and, importantly, a clear underrepresentation of employed women. Thus, larger, better characterized studies are needed.

In this study, we set out to test prospectively the association between effort–reward imbalance and later coronary heart disease in a uniquely large multicohort dataset with individual-participant data from 11 cohort studies. In doing so, we use predefined harmonized assessments of effort–reward imbalance, job strain, and coronary heart disease events^3,17^ and took into account the role of job strain in potentially generating a link between effort–reward imbalance and coronary heart disease.

## METHODS

### Study Population

We used data from 11 independent cohort studies initiated between 1985 and 2005 in Denmark, Finland, France, Germany, Sweden, and the United Kingdom. All studies were part of the “individual-participant data meta-analysis in working populations” (IPD-Work) consortium that was established at the four centers initiative meeting in 2008.^3^ The aim of the consortium is to provide a large-scale harmonized data base for the longitudinal estimation of associations of predefined psychosocial working conditions with disease outcomes. The participating studies comply with the Declaration of Helsinki and were approved by the local review boards. Informed consent has been obtained from participants. Details of the design and participants in the studies included in the present analysis are provided in eAppendix S1; http://links.lww.com/EDE/B193. Studies are briefly identified, and their names spelled out, in Table 1.

Our analyses were based on participants who were employed at the time of the baseline assessment in each cohort study. Participants with missing data on age, sex, effort–reward imbalance, or incident coronary heart disease and those with a prevalent coronary heart disease diagnosis at baseline were excluded from the analyses (N excluded = 6,047; eAppendix S1; http://links.lww.com/EDE/B193). This resulted in an analytical sample of 90,164 employed people, who were followed up for a mean of 9.8 years.

### Assessment of Effort–Reward Imbalance

Effort–reward imbalance was measured using a range of questionnaire items. The availability of items differed between the studies: the original effort–reward questionnaire^18^ was included in three studies (Gazel, HNR, and WOLF-N), whereas eight other studies used abbreviated versions. Therefore, a common measurement approach was developed, harmonized, and validated across the constituent studies. A detailed description of the scale construction, harmonization, and validation process has been provided.^17^ Importantly, this process was completed before conducting any exposure–outcome analyses; that is, investigators who performed the validation of the partial proxy scales were blinded to any information about incident coronary heart disease.

In the effort–reward imbalance questionnaire, participants were asked to respond to a range of enquiries regarding psychosocial aspects of their job. For each participant, mean response scores were calculated for effort items (i.e., questions about work demands and efforts) and reward items (questions about monetary and nonmonetary rewards at work). The Pearson correlation coefficients between the harmonized scales used in this study and the complete versions were high, being *r* > 0.92 for the effort scales and *r* > 0.75 for the reward scales.^17^

We calculated the effort–reward imbalance score as the ratio of the harmonized effort scale divided by the harmonized reward scale.^17^ Values >1 indicate that effort exceeds reward (an effort–reward imbalance). The score was then dichotomized (effort–reward imbalance versus no effort–reward imbalance).

### Ascertainment of Coronary Heart Disease

We ascertained information on incident coronary heart disease during the follow-up period from national hospitalization and death registries in all studies except Gazel and HNR in which hospitalization registry data were not available and nonfatal events were based on self-report in annually distributed questionnaires. All nonfatal events in HNR study were additionally checked against medical records. We used date of diagnosis, hospital admission due to myocardial infarction, or coronary heart disease death to define coronary heart disease incidence, coded using International Classification of Diseases (ICD) codes^19^ or MONICA definition.^20^ In the mortality and hospital records, we used only the main diagnosis. We included all nonfatal myocardial infarctions that were recorded as I21–I22 (ICD-10) or 410 (ICD-9) and coronary deaths recorded as I20–I25 (ICD-10) or 410–414 (ICD-9).

### Covariates

We used age, sex, socioeconomic position, lifestyle-related factors, and job strain as covariates because they are potentially related to both effort–reward imbalance and the risk of coronary heart disease. Socioeconomic position was based on occupational title obtained from employers or national registers (COPSOQ-I, COPSOQ-II, DWECS, FPS, IPAW, and PUMA) or participant-completed questionnaires (in Whitehall II, HNR, WOLF-F, and WOLF-S). The harmonized socioeconomic position measure was categorized into low, intermediate, high, and other. Participants with missing information on job title or who were self-employed were included in the “other” category.

We extracted the lifestyle-related factors tobacco smoking, alcohol intake, and leisure-time physical activity from participant-completed questionnaires in all studies. Smoking was classified into never, former, or current smokers.^21^ We used responses to questions on the total number of alcoholic drinks consumed in a week to classify the participants as nondrinkers, moderate drinkers (women: 1–14 drinks/week, men: 1–21 drinks/week), high-intermediate drinkers (women: 15–20 drinks/week, men: 22–27 drinks/week), and heavy drinkers (women: ≥21 drinks/week, men: ≥28 drinks/week).^22^ Body mass index (BMI, weight in kilograms divided by height in meter squared) was calculated using data on height and weight, which were self-reported in seven studies (COPSOQ-I, COPSOQ-II, DWECS, FPS, IPAW, and PUMA) and measured directly in four studies (HNR, Whitehall II, WOLF-N, and WOLF-S). We categorized BMI according to the World Health Organization recommendations: <18.5 kg/m^2^ (underweight), 18.5–24.9 kg/m^2^ (normal weight), 25–29.9 kg/m^2^ (overweight), and ≥30 kg/m^2^ (obese). Participants with BMI values <15 or >50 were excluded from the analysis including BMI because of suspected measurement error.^23^ We classified participants into three categories according to their leisure-time physical activity level (passive, moderately active, highly active).^24^

Finally, we ascertained job strain from the participants’ responses to questions on demands and control aspects of their work at study baseline.^3,9^ The responses were scored and for each participant, mean scores were calculated for job demand items and job control items. Based on the study-specific medians, participants’ job demands and control were defined as high or low. A combination of high demands and low control was defined as job strain and all other combinations as no job strain.

### Statistical Analyses

The study-specific time-dependent interaction terms between effort–reward imbalance and logarithm (follow-up period) were all nonsignificant suggesting the proportional hazards assumption had not been violated. Accordingly, the associations between effort–reward imbalance and incident coronary heart disease were analyzed in each study using Cox proportional hazards regression models. Each participant was followed up from the date of their effort–reward imbalance assessment to the earliest of the following: coronary heart disease event, noncoronary death, or the end of follow-up. The minimal statistical model included age and sex as covariates. The maximal statistical model also contained socioeconomic position, lifestyle-related risk factors (physical activity, smoking, alcohol intake, BMI), and job strain. In additional analysis, we examined the associations of efforts and rewards (both dichotomized at median) with coronary heart disease.

We pooled the study-specific effect estimates and their standard errors in a random effects meta-analysis, which provides a conservative estimate of an association. Heterogeneity in the effect estimates was quantified using the *I*^2^ statistic, which indicates the proportion of the total variation in the estimates that is due to between-studies variation. As sensitivity analyses, we repeated the analysis after excluding coronary heart disease events during the first 3 years of follow-up (to explore reverse causality) and in samples stratified by sex, age, and socioeconomic position.

We used the SAS 9.4 (Cary, NC) statistical software to analyze study-specific data. Meta-analyses were performed using Stata MP 13.1, metan package (Stata Corporation, College Station, TX).

## RESULTS

In 90,164 participants included in the analysis, mean age at study entry was 45.1 (SD = 8.6) years and 60.8% were women (Table 1). The proportion of individuals with effort–reward imbalance varied between 8% and 51% depending on the study, which may reflect both true differences in prevalence and differences in the measures of effort–reward imbalance despite harmonization. It was 31.7% in the total population.

**TABLE 1.**
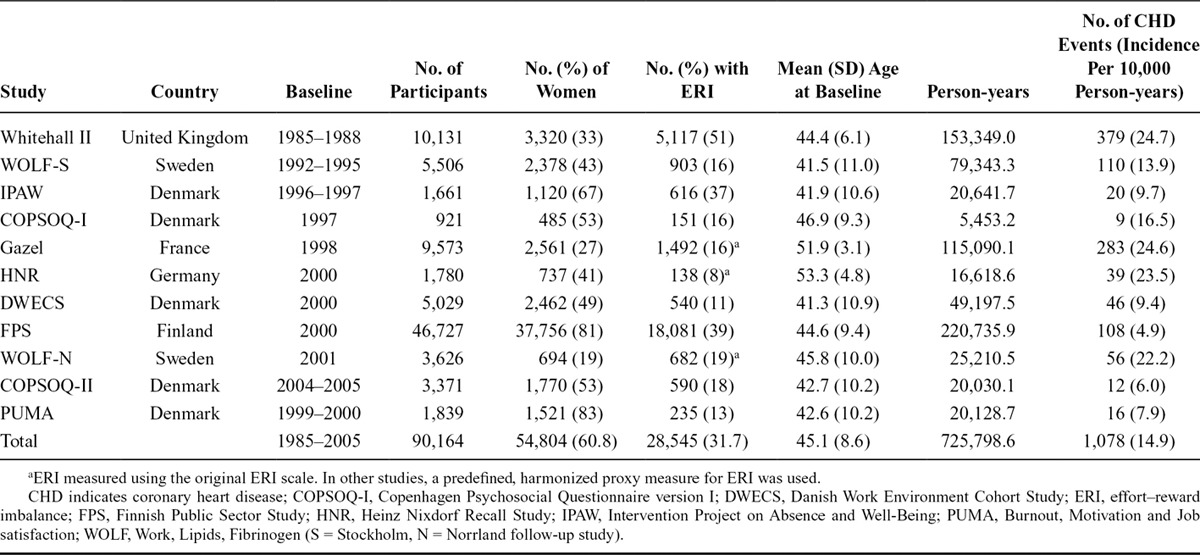
Characteristics of Study Participants in 11 European Cohort Studies (1985–2010)

### Effort–Reward Imbalance and Incident Coronary Heart Disease

One of the 11 studies included in this meta-analysis has previously reported on the association between effort–reward imbalance and incident coronary heart disease (nonfatal myocardial infarction or coronary death).^13^ In the Whitehall II study, the hazard ratio for effort–reward imbalance was 1.28 (95% confidence interval [CI] = 0.89, 1.84).^13^

In the present meta-analysis, 1,078 incident coronary heart disease events were recorded during a total of 725,799 person-years at risk (mean follow-up 9.8 years). In an analysis in which hazard ratios were adjusted for age and sex, effort–reward imbalance was associated with a 1.16-fold increased hazard of incident coronary heart disease (summary hazard ratio across studies: 1.16 (95% CI = 1.01, 1.34; Figure 1). There was no apparent heterogeneity in study-specific estimates (*I*^2^ = 0%; *P* = 0.89) in 8 of 11 studies, the hazard ratio exceeded one although in all studies CIs were wide. The pooled hazard ratio was unchanged after further adjustment for socioeconomic position, lifestyle factors, and job strain (1.16; 95% CI = 1.00, 1.35; Table 2; estimates for covariates are provided in eAppendix S2; http://links.lww.com/EDE/B193). The association was marginally strengthened after exclusion of coronary heart disease cases that occurred during the first 3 years of follow-up (age- and sex-adjusted hazard ratio: 1.21 (95% CI = 1.03, 1.41; eAppendix S3; http://links.lww.com/EDE/B193). In the subgroup analyses (eAppendix S4; http://links.lww.com/EDE/B193), the association appeared to be more pronounced in younger participants (<50 years) and in participants in a lower socioeconomic position, but a test for heterogeneity suggests that the observed differences between subgroups were small (*I*^2^ for socioeconomic status = 20.8%; *P* = 0.28; *I*^2^ for age = 72.4%; *P* = 0.06).

**TABLE 2.**
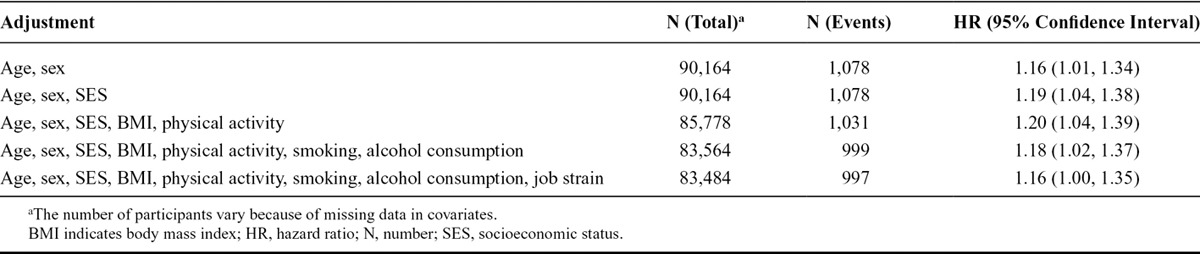
Summary Hazard Ratios from Random Effects Meta-analysis of Serially Adjusted Association Between Effort–Reward Imbalance and Incident Coronary Heart Disease (1985–2010)

**FIGURE 1.**
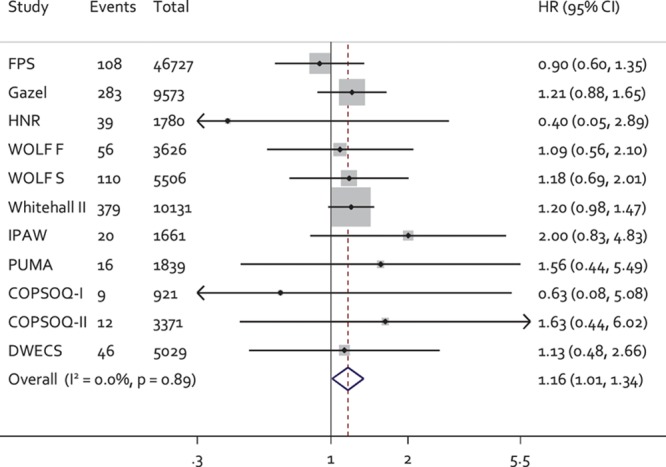
Age- and sex-adjusted HRs from random effects meta-analysis of the association between effort–reward imbalance and incident coronary heart disease (1985–2010; *P* value for test of heterogeneity). HR indicates hazard ratio.

In analysis of the components of effort–reward imbalance, the age- and sex-adjusted hazard ratio of incident coronary heart disease was 0.99 (95% CI = 0.87, 1.13) for high (above median) versus low (median or below) efforts and 1.18 (95% CI = 1.04, 1.33) for low (below median) versus high (median or higher) rewards.

### Combined Effect of Effort–Reward Imbalance and Job Strain

Of the 90,052 participants with data on job strain, 8,797 (9.8%) reported both effort–reward imbalance and job strain, 25,219 (28.0%) reported either effort–reward imbalance or job strain, but not both, and 56,036 (62.2%) were unexposed to these two work-related stressors (cross-tabulation of effort–reward imbalance and job strain overall and in primary studies in eAppendix S5; http://links.lww.com/EDE/B193). Figure 2 suggests that the effect of effort–reward imbalance and job strain on incident coronary heart disease is additive. Thus, the age- and sex-adjusted hazard ratio for coronary heart disease (compared with neither effort–reward imbalance nor job strain) was 1.16 (95% CI = 1.01, 1.34) for one work stressor (either job strain or effort–reward imbalance, but not both) and 1.41 (1.12–1.76) for two work stressors (both job strain and effort–reward imbalance). Heterogeneity in study-specific estimates was low (*I*^2^ for one stressor = 21.1%; *P* = 0.25, *I*^2^ for two stressors = 0%; *P* = 0.84).

**FIGURE 2.**
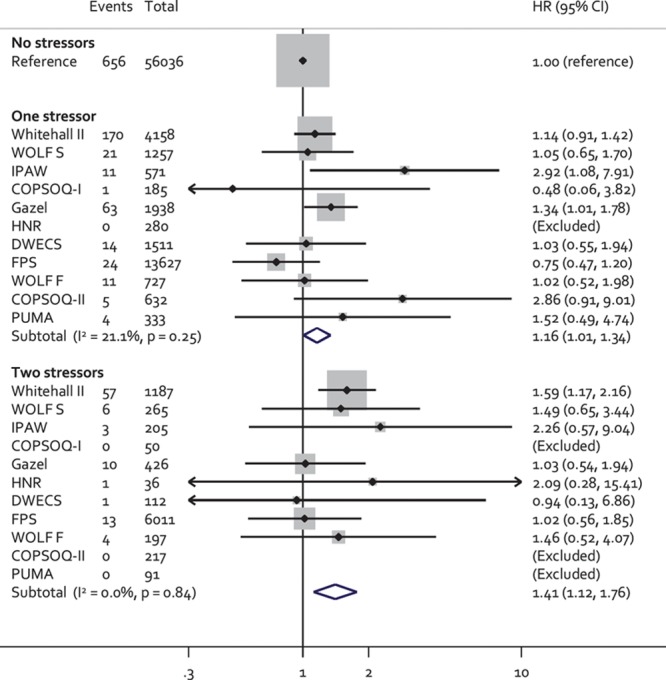
Age- and sex-adjusted HRs for the association of 1 and 2 stressors (effort–reward imbalance and job strain) with incident coronary heart disease (1985–2010). HR indicates hazard ratio.

## DISCUSSION

In this collaborative meta-analysis of individual-participant data from 11 independent cohort studies from Europe, perceived effort–reward imbalance at work was associated with an elevated hazard of coronary heart disease. This association persisted after adjusting for sociodemographic and behavioral coronary heart disease risk factors and also “job strain,” a complementary established measure of stressful work.

That the effect estimates did not substantially change after these statistical adjustments suggests that effects of effort–reward imbalance on health risk behaviors are not a major mechanism linking our exposure and outcome.^25^ A second plausible explanation is via a psychobiological stress reaction unmeasured in the present studies. Thus, effort–reward imbalance at work might be associated with biologic changes that are known risk factors for coronary heart disease,^26,27^ such as elevated fibrinogen and atherogenic blood lipids,^28^ elevated blood pressure,^29^ reduced heart rate variability,^30^ elevated inflammatory markers,^31,32^ and dysregulation of the hypothalamic–pituitary–adrenal axis.^33^ Many of these biological effects may arise as a functional adaptation to excessive hypothalamic pituitary adrenal and sympathetic nervous system stimulation that can be regarded as a consequence of sustained allostatic load.^34^

Our subgroup analyses were hampered by low power and were not all hypothesis driven. However, the findings raised the possibility that the association between effort–reward imbalance and coronary heart disease may be stronger among employees with low compared with high socioeconomic position, and in younger than older participants. That effort–reward imbalance might be more health hazardous in employees of lower socioeconomic position accords with other findings.^13,35^ It could be that people with a low socioeconomic position have additional risk factors and fewer resources and are therefore more susceptible to the adverse health effects of work-related stress. We are unclear why there might be effect modification by age, although effort–reward imbalance at younger ages might mark a longer exposure period than effort–reward imbalance at older ages.

The association between effort–reward imbalance and coronary heart disease was independent of job strain. Our findings support the view that effort–reward imbalance and job strain represent complementary models of work stress, capturing different aspects of a stressful work environment and that their effects on coronary heart disease are additive. Individuals with one of the two work stressors (but not both) had a 16% higher hazard ratio of incident coronary heart disease compared with those free of these stressors; in individuals with both work stressors, this hazard ratio was 41% higher. Although the estimates for the single measures suggest a small increase in the hazard of disease, public health impact may be high owing to the high number of exposed individuals. Our observation of an additive effect of two distinct aspects of work-related stress (i.e., effort–reward imbalance and job strain) further justifies this notion.

Our study adds to a growing body of evidence demonstrating a link between adverse psychosocial work environment and coronary heart disease.^2,3^ However, at least three limitations of our study merit attention. First, the exposure was only measured once at baseline while repeated assessment of this time-varying characteristic would have been optimal. Misclassification of de facto unexposed participants as exposed may have occurred as a single-point measurement does not differentiate between short-term episodes of high work load and potentially harmful chronic conditions. Moreover, not all studies used the psychometrically validated original version of the effort–reward imbalance questionnaire, leading to large variation in the prevalence of effort–reward imbalance between studies. However, the associations with coronary heart disease did not differ between studies using the original effort–reward questionnaire (hazard ratio for exposure versus no exposure to effort–reward imbalance: 1.16) and those using a proxy measure (hazard ratio: 1.17).

Second, although our exposure measure preceded the outcome, we cannot rule out bias due to reverse causation specifically if incident events occurred in short time after exposure assessment, because people may spontaneously reduce efforts at work in the years before cardiac events in response to symptoms of disease. However, a major bias is unlikely because the results were strengthened rather than weakened in sensitivity analyses excluding events that occurred in the first 3 years of follow-up. Furthermore, we measured a limited set of socioeconomic and lifestyle covariates^36,37^ and the measurement was restricted to baseline only. Thus, our data do not allow a determination of whether the covariates were likely to represent mediators or confounding factors. These limitations may have contributed to an over- or underestimation of the association between effort–reward imbalance and incident coronary heart disease.

It is also important to consider that the dataset was restricted to European countries with high occupational safety and health standards including distinct antistress policies. The generalizability to other regions and cultures is therefore unclear. We have recently shown that the prevalence as well as the health effects of work stress may vary across different countries and between different types of welfare state regimes: countries with more generous social security systems and an active labor market policy had a lower prevalence of health adverse psychosocial working conditions^38^ and showed a less pronounced association between work stress and depressive symptoms, a predictor of coronary heart disease.^39^ As the studies included in this meta-analysis were mainly from Northern and Western Europe, we cannot rule out that associations between effort–reward imbalance and coronary heart disease may be different among working people from countries with less active welfare policy and fewer measures of occupational health protection. Further research including cohorts from other parts of Europe and other continents is therefore warranted.

These limitations have to be balanced against some specific strengths of the IPD-Work consortium study. A major advantage is the large number of longitudinal observations that allows, for the first time, for precise estimation of the relationship between effort–reward imbalance and coronary heart disease as well as subgroup analyses to assess effect modification. The large sample covering white- and blue-collar employees from different European countries supports the notion that findings can be generalized beyond the scope of a single study or occupational group. Importantly, both sexes were also well represented. The fact that the chosen operational definition of effort–reward imbalance was predefined and published before the analysis of empirical outcome data^17^ is a further strength of this study. The availability of alternative ways of defining effort–reward imbalance can encourage multiple testing and result in bias because of selective reporting (i.e., selective revealing or suppression of information by the investigators). The predefined operationalizations in our study reduced the likelihood of such post hoc decisions.

In conclusion, the results of this multicohort study suggest that “effort–reward imbalance” defines a specific work-related risk constellation for coronary heart disease that is independent of job strain. Reliable scientific evidence based on theoretical models is valuable for disease prevention because it can guide systematic efforts to eliminate important sources of work-related stress and thus potentially reduce the burden of coronary heart disease in the working population.^40^ The present observational results suggest that preventive measures and policies that address the imbalance between high efforts and low rewards might reduce disease incidence, especially when combined with approaches designed to prevent job strain.

Further research is needed to evaluate the benefits and harms of monitoring stressful work environments in a systematic and regular way at the company level and implementation of measures to reduce any adverse impact of detected effort–reward imbalance and job strain.^41,42^ Examples of such interventions include securing fair wage and promotion opportunities, developing a culture of recognition and supportive leadership, reducing excessive workload and working hours, and improving control and autonomy at work. To facilitate sustainability, these measures may need to be reinforced by active labor market policies at the national and international levels. Future registered cluster-randomized trials and natural experiments assessing the additional value of such workplace intervention strategies compared with usual coronary heart disease prevention that emphasizes conventional risk factors modification (smoking cessation, healthy diet, physical activity, antihypertensive treatment, and statin therapy) would be particularly informative.

## Supplementary Material
