# Supplementary material for: Effort–Reward Imbalance at Work and Incident Coronary Heart Disease: A Multicohort Study of 90,164 Individuals
Source: Epidemiology. 2017 Jun 1;28(4):619–26. doi: 10.1097/EDE.0000000000000666 (PMC5457838; doi:10.1097/EDE.0000000000000666)
Supplement: Supplementary file 1 [file ede-28-619-s001.pdf]

eAppendix S1. Studies and participants

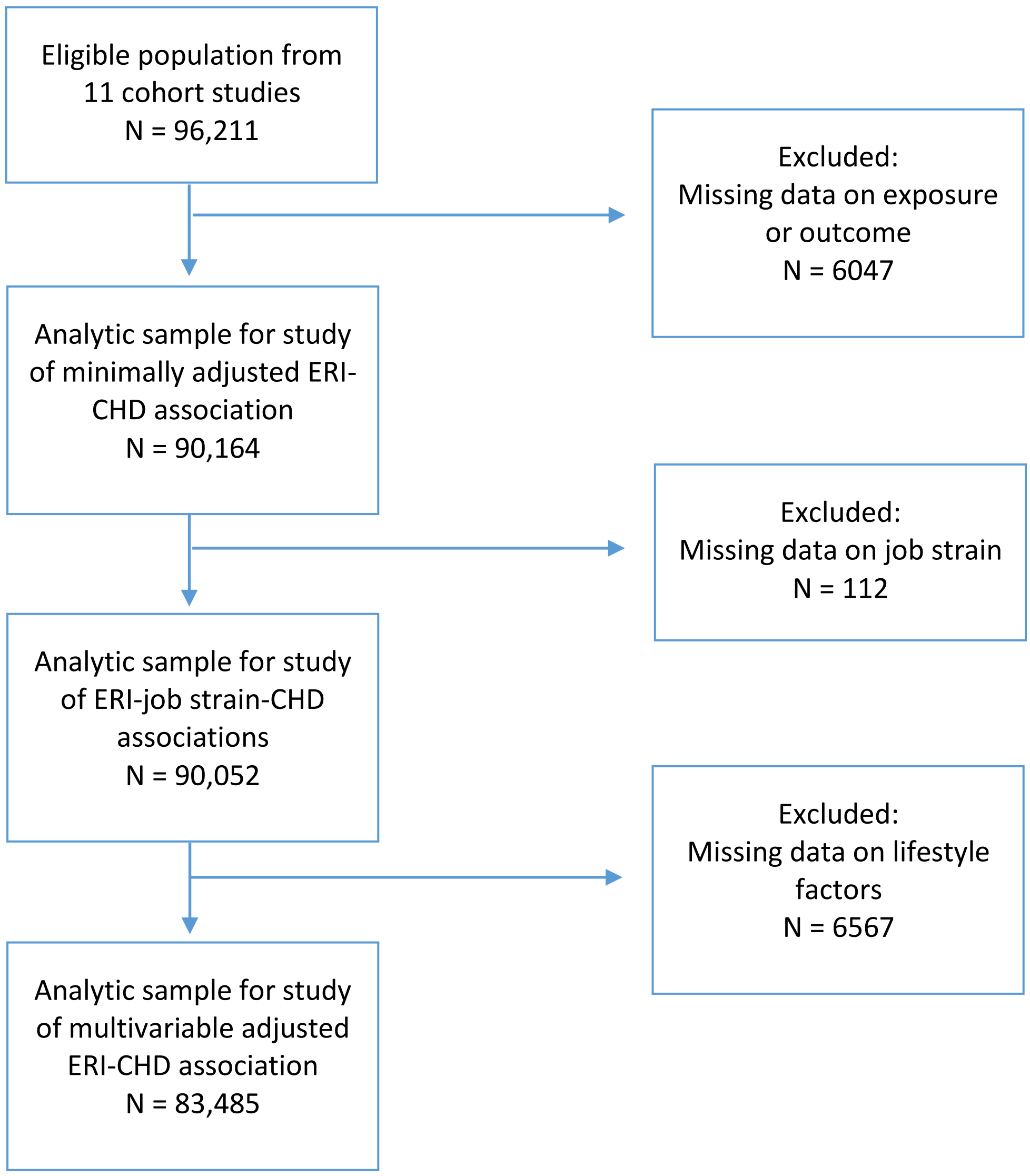

Figure S1. Flowchart of the selection of the analyzed samples.

We included all the 8 original cohort studies from the IPD-Work job strain meta-analysis (Kivimaki et al. Lancet 2012; 380(9852):1491-7. PMID: 22981903) which additionally had data on effort-reward imbalance: COPSOQ-1, DWECS, FPS, GAZEL, IPAW, Whitehall II, WOLF-S, and WOLF-N. Since 2012, the IPD-Work consortium has expanded to include 3 new cohort studies with data on effort-reward imbalance, job strain and coronary heart disease: COPSOQ-II, HNR, PUMA. These studies were also included. For 5 studies in the job strain meta-analysis, data on effort-reward imbalance were not available: BELSTRESS, HeSSup,

POLS, Still Working, NWCS. Thus, those studies were not included in the present meta-analysis. A brief description of the 11 included cohort studies are provided below:

Copenhagen Psychosocial Questionnaire version I (COPSOQ-I)

The COPSOQ-I is a prospective cohort study of a random sample of Danish residents selected from the Danish population register. The participants were aged 20-60 years of age and were in paid employment at the study baseline in 1997. A baseline questionnaire and an invitation to take part was posted to 4 000 people and 2 454 individuals agreed to participate [1]. In Denmark, questionnaire- and register-based studies do not require approval from the Danish National Committee on Biomedical Research Ethics (Den Centrale Videnskabetiske komité). COPSOQ-I was approved by and registered with the Danish Data protection agency (registration number: 2008 - 54 - 0553).

Copenhagen Psychosocial Questionnaire version II (COPSOQ-II)

COPSOQ-II was carried out in 2004-2005. It included a follow up of respondents from COPSOQ I and also a representative sample of Danish residents aged 20-60 at study baseline. The questionnaire was sent to 8 000 individuals from the random sample and 4 732 individuals responded. The questionnaire could be completed using the posted questionnaire or via internet [2]. In Denmark, questionnaire- and register-based studies do not require ethics committee approval. COPSOQ-II was approved by and registered with the Danish Data protection agency (registration number: 2004-54-1493).

Danish Work Environment Cohort Study (DWECS)

DWECS is a split panel survey of working age Danish people. The cohort was established in 1990, when a simple random sample of men and women, aged 18-59, was drawn from the Danish population register. The participants have been followed up at five year intervals and data from the year 2000 was used for the IPD-Work. That year 11 437 individuals were invited to participate and 8 583 agreed to do so [3, 4]. In Denmark, questionnaire- and register-based studies do not require ethics committee approval. DWECS was approved by and registered with the Danish Data protection agency (registration number: 2007-54-0059).

Finnish Public Sector study (FPS)

The Finnish Public Sector study is a prospective cohort study comprising the entire public sector personnel of 10 towns (municipalities) and 21 hospitals in the same geographical areas. Participants, recruited from employers' records in 2000-2002, were individuals who were employed in the study organisations at the time of the questionnaire survey [5]. 48 592 individuals (9 337 men and 39 255 women aged 17 to 65) responded to the questionnaire. Ethical approval was obtained from the ethics committee of the Finnish Institute of Occupational Health.

Gazel

Gazel is a prospective cohort study of 20 625 employees (15 011 men and 5 614 women) of France's national gas and electricity company, Electricité de France-Gaz de France (EDF-GDF) [6, 7] . Since the study baseline in 1989, when the participants were aged 35–50 years, they have been posted an annual follow-up questionnaire to collect data on health, lifestyle, individual, familial, social, and occupational factors. Effort-reward imbalance was measured in Gazel in 1998, which we treated as a baseline year for our analyses. The GAZEL study received approval from the national commission overseeing ethical data collection in France (Commission Nationale Informatique et Liberté).

Heinz Nixdorf Recall study (HNR)

The Heinz Nixdorf Recall Study is a prospective population-based cohort study of individuals randomly selected from the mandatory lists of residence in the metropolitan Ruhr area in Germany. Details of the study methods have been described previously [8, 9] . Briefly, 4 814 participants aged 45-75 years were enrolled at study baseline in 2000-2003. Job stress measures and comprehensive medical data were collected during the baseline examination. The HNR was approved by the institutional local ethical committees and a quality management system according to European industrial norms (DIN EN ISO 9001:2000) was applied

Intervention Project on Absence and Well-being (IPAW)

IPAW is a 5-year psychosocial work environment intervention study including 22 intervention and 30 control work places in three organisations (a large pharmaceutical company, municipal technical services and municipal nursing homes) in Copenhagen, Denmark [10, 11]. The baseline questionnaire was posted to all the employees at the selected work-sites between 1996 and 1997. Of the 2 721 employees who worked at the 52 IPAW sites, 2 068 men and women completed the baseline questionnaire. Interventions took place at 22 workplaces during 1996-98 at the organisational and interpersonal level. IPAW was approved by and registered with the Danish Data Protection Agency (registration number: 2000-54-0066).

Burnout, Motivation and Job Satisfaction study (Danish acronym: PUMA)

Burnout, Motivation and Job Satisfaction study (Danish acronym: PUMA) is an intervention study of burn-out among employees in the human service sector [12]. Selection criteria for the participating organisations was that they had between 200 and 500 employees, that occupational groups within each organisation were willing to participate and that the organisations would commit to the entire five-year study period. Participants gave consent to having their national identity numbers collected and used in later record linkages to Danish hospitalisation and cause of death registries (Hospitalsindlæggelsesregisteret, Dødsårsagsregisteret. At study baseline in 1999-2000, 1 914 participants agreed to take part. PUMA was approved by the Scientific Ethical Committees (Videnskabsetisk Komiteer) in the counties in which the study was conducted and approved by and registered with the Danish Data Protection Agency (registration number: 2000-54-0048).

Whitehall II

The Whitehall II study is a prospective cohort study set up to investigate socioeconomic determinants of health. At study baseline in 1985-1988, 10 308 civil service employees (6 895 men and 3 413 women) aged 35-55 and working in 20 civil service departments in London were invited to participate in the study [13]. The Whitehall II study protocol was approved by the University College London Medical School committee on the ethics of human research. Written informed consent was obtained at each data collection wave.

WOLF (Work, Lipids, and Fibrinogen) Stockholm and WOLF Norrland studies

The WOLF (Work, Lipids, and Fibrinogen) Stockholm study is a prospective cohort study of 5 698 people (3 239 men and 2 459 women) aged 19–70 and working in companies in Stockholm county [14]. WOLF Norrland is a prospective cohort of 4 718 participants aged 19-65 working in companies in Jämtland and Västernorrland counties [15]. At study baseline the participants underwent a clinical examination and completed a set of health questionnaires. For WOLF Stockholm, the baseline assessment was undertaken at 20 occupational health units between November 1992 and June 1995 and for WOLF Norrland at 13 occupational health service units in 1996-98. In 2000-2003, 3 630 people from the WOLF Norrland study were participating in the WOLF Norrland Follow-up study (WOLF F), where also 1 706 new participants responded to the questionnaire [16]. From WOLF Norrland, only data from the follow-up study are included in effort-reward imbalance analyses. The Regional Research Ethics Board in Stockholm, and the ethics committee at Karolinska Institutet, Stockholm, Sweden approved the study.

References

1. Kristensen, T.S., et al., *The Copenhagen Psychosocial Questionnaire--a tool for the assessment and improvement of the psychosocial work environment.* Scand J Work Environ Health, 2005. **31**(6): p. 438-49.
2. Pejtersen, J.H., et al., *The second version of the Copenhagen Psychosocial Questionnaire.* Scand J Public Health, 2010. **38**(3 Suppl): p. 8-24.

3. Burr, H., et al., *Trends in the Danish work environment in 1990–2000 and their associations with labor-force changes.* Scand J Work Environ Health, 2003. **29**(4): p. 270-279.
4. Feveile, H., et al., *Danish Work Environment Cohort Study 2005: From idea to sampling design.* Statistics in Transition, 2007. **8**(3): p. 441-458.
5. Kivimäki, M., et al., *Socioeconomic Position, Co-Occurrence of Behavior-Related Risk Factors, and Coronary Heart Disease: the Finnish Public Sector Study.* Am J Public Health, 2007. **97**(5): p. 874-879.
6. Goldberg, M., et al., *Cohort profile: the GAZEL Cohort Study.* Int J Epidemiol, 2007. **36**(1): p. 32-9.
7. Zins, M., A. Leclerc, and M. Goldberg, *The French GAZEL Cohort Study: 20 Years of Epidemiologi REsearch.* Advances in Life Course Research, 2009. **14**: p. 135-146.
8. Schmermund, A., et al., *Assessment of clinically silent atherosclerotic disease and established and novel risk factors for predicting myocardial infarction and cardiac death in healthy middle-aged subjects: rationale and design of the Heinz Nixdorf RECALL Study. Risk Factors, Evaluation of Coronary Calcium and Lifestyle.* Am Heart J, 2002. **144**(2): p. 212-8.
9. Stang, A., et al., *Baseline recruitment and analyses of nonresponse of the Heinz Nixdorf Recall Study: identifiability of phone numbers as the major determinant of response.* Eur J Epidemiol, 2005. **20**(6): p. 489-96.
10. Nielsen, M., T. Kristensen, and L. Smith-Hansen, *The Intervention Project on Absence and Well-being (IPAW): design and results from the baseline of a 5-year study.* Work and Stress, 2002. **16**: p. 191-206.
11. Nielsen, M.L., et al., *Impact of the psychosocial work environment on registered absence from work: a two-year longitudinal study using the IPAW cohort.* Work & Stress 2004. **18**(4): p. 323-335.
12. Borritz, M., et al., *Burnout among employees in human service work: design and baseline findings of the PUMA study.* Scand J Public Health, 2006. **34**(1): p. 49-58.
13. Marmot, M.G., et al., *Health inequalities among British civil servants: the Whitehall II study.* Lancet, 1991. **337**(8754): p. 1387-93.
14. Peter, R., et al., *High effort, low reward, and cardiovascular risk factors in employed Swedish men and women: baseline results from the WOLF Study.* J Epidemiol Community Health, 1998. **52**: p. 540-547
15. Alfredsson, L., et al., *Job strain and major risk factors for coronary heart disease among employed males and females in a Swedish study on work, lipids and fibrinogen.* Scand J Work Environ Health, 2002. **28**(4): p. 238-48.
16. Åkerstedt, T., et al., *Predicting changes in sleep complaints from baseline values and changes in work demands, work control, and work preoccupation--the WOLF-project.* Sleep Med. **13**(1): p. 73-80.

eAppendix S2. Summary hazard ratios for effort-reward imbalance and covariates as predictors of incident coronary heart disease

Baseline characteristic	Age- and sex-adjusted		Fully (mutually) adjusted	
	Hazard ratio	95% CI	Hazard ratio	95% CI
**Effort-reward imbalance**				
No	1.00		1.00	
Yes	1.16	1.01-1.34	1.16	1.00-1.35
**Job strain**				
No	1.00		1.00	
Yes	1.25	1.05-1.48	1.11	0.92-1.34
**Age (per year)**	1.10	1.09-1.11	1.10	1.08-1.11
**Sex**				
Women	1.00		1.00	
Men	4.20	2.96-5.94	4.38	3.25-5.91
**SES**				
Other	0.91	0.47-1.76	0.96	0.49-1.89
Low	1.00		1.00	
Intermediate	0.86	0.69-1.06	0.97	0.75-1.25
High	0.58	0.48-0.69	0.72	0.58-0.88
**Physical activity**				
Low	1.00		1.00	
Intermediate	0.75	0.61-0.92	0.83	0.69-1.00
High	0.61	0.48-0.78	0.72	0.52-0.98
**Smoking**				
Never	1.00		1.00	
Ex	1.15	0.94-1.42	1.16	0.95-1.43
Current	2.11	1.81-2.47	2.15	1.83-2.54
**Alcohol use**				
None	1.55	1.28-1.87	1.46	1.22-1.75
Moderate	1.00		1.00	
Intermediate	1.00	0.76-1.33	0.96	0.72-1.27
Heavy	1.20	0.98-1.47	1.03	0.83-1.28
**BMI**				
Underweight	1.37	0.51-3.72	1.03	0.33-3.27
Healthy weight	1.00		1.00	
Overweight	1.42	1.24-1.63	1.35	1.15-1.58
Obese, class I	2.11	1.66-2.67	1.94	1.45-2.59
Obese, class II	3.22	2.15-4.81	2.71	1.76-4.18
Obese, class III	3.89	1.42-10.64	3.11	1.13-8.61

eAppendix S3. Association between effort-reward imbalance and incidence coronary heart disease after exclusion of the first three years of follow-up

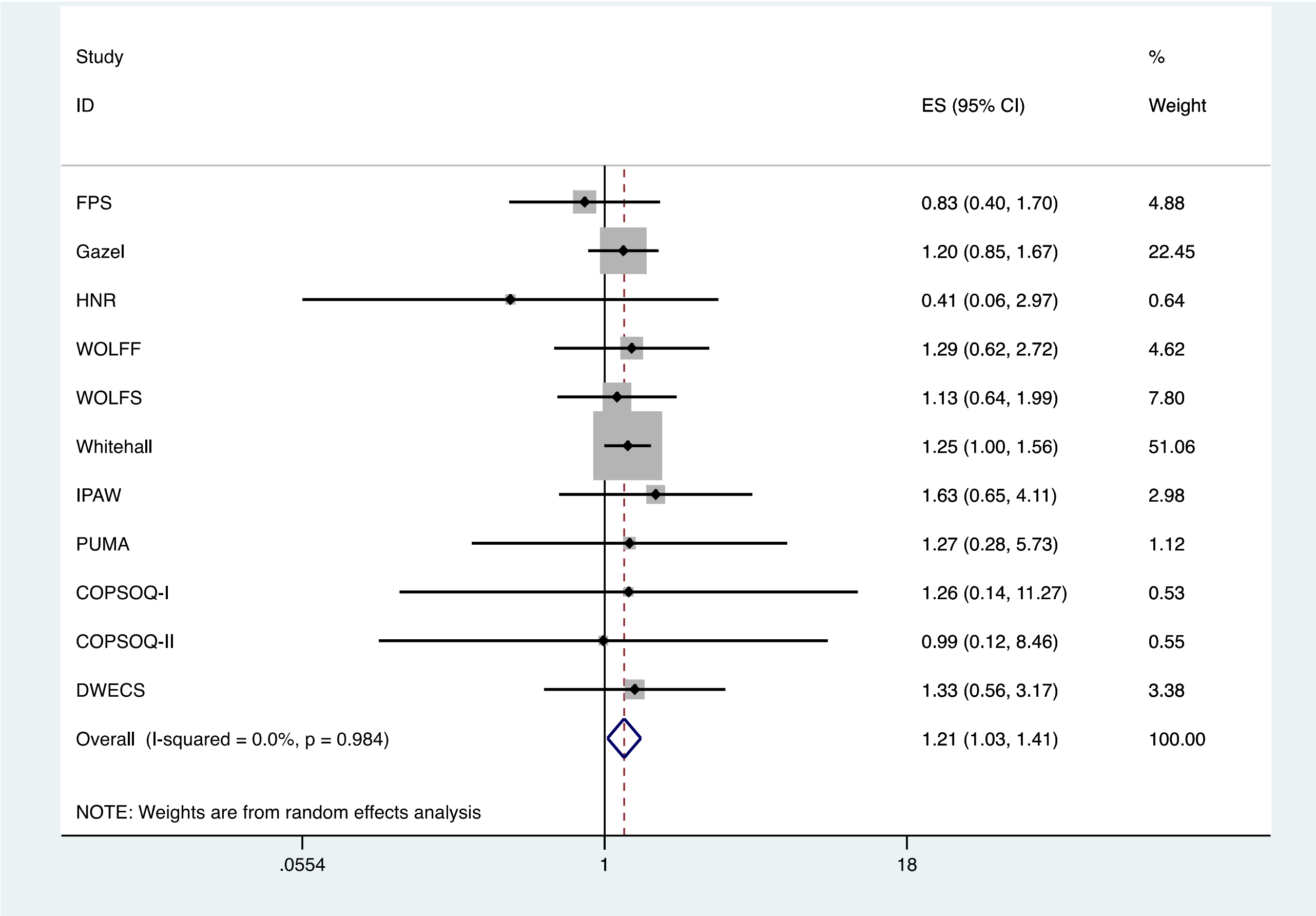

eAppendix S4. Hazard ratios from random-effects meta-analysis of the association between effort-reward imbalance and incident coronary heart disease in relation to sex, age, socioeconomic position and type of effort-reward imbalance (ERI) questionnaire (adjustment for age and sex where relevant) Some cohorts were excluded because no participant in the exposure group experienced coronary heart disease.

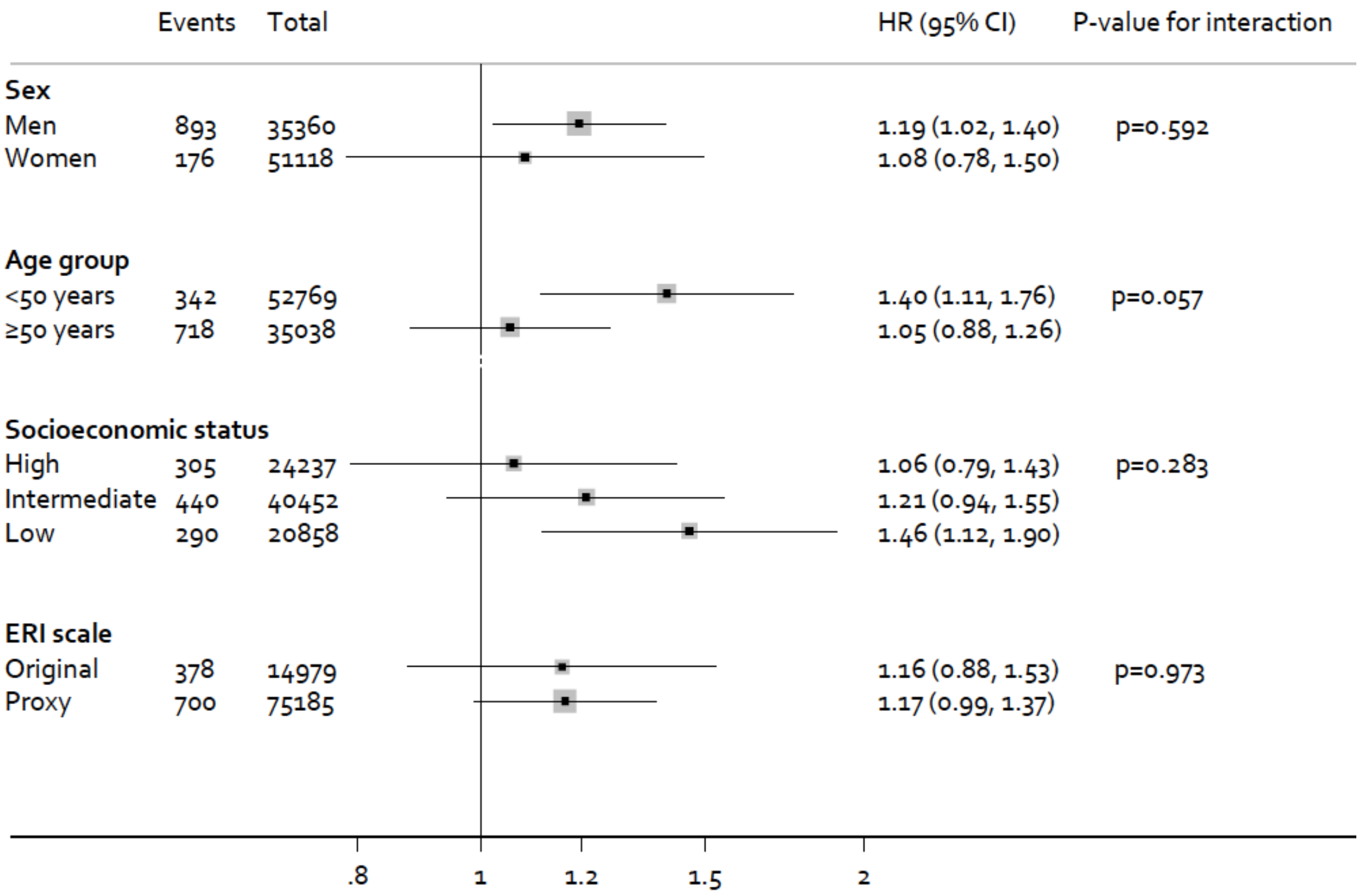

eAppendix S5. Association between effort-reward imbalance and job strain overall and in primary studies

Table S4a. Numbers (%) of participants with effort-reward imbalance and job strain in a pooled sample of 11 studies.

	**Effort-reward imbalance**		
**Job strain**	No	Yes	Total
No	56,036 (62.2%)	19,721 (21.9%)	75,757 (84.1%)
Yes	5498 (6.1%)	8797 (9.8%)	14,295 (15.9%)
Total	61,534 (68.3%)	28,518 (31.7%)	90,052 (100%)

Table S4b. Numbers (%) of participants with effort-reward imbalance and job strain in COPSOQ I.

	**Effort-reward imbalance**	
**Job strain**	No	Yes
No	686 (74.5)	101 (11.0)
Yes	84 (9.1)	50 (5.4)

Table S4c. Numbers (%) of participants with effort-reward imbalance and job strain in COPSOQ II.

	**Effort-reward imbalance**	
**Job strain**	No	Yes
No	2520 (74.8)	373 (11.1)
Yes	259 (7.7)	217 (6.4)

Table S4d. Numbers (%) of participants with effort-reward imbalance and job strain in DWECS.

	**Effort-reward imbalance**	
**Job strain**	No	Yes
No	3406 (67.7)	428 (8.5)
Yes	1083 (21.5)	112 (2.2)

Table S4e. Numbers (%) of participants with effort-reward imbalance and job strain in FPS.

	**Effort-reward imbalance**	
**Job strain**	No	Yes
No	27062 (57.9)	12055 (25.8)
Yes	1572 (3.4)	6011 (12.9)

Table S4f. Numbers (%) of participants with effort-reward imbalance and job strain in GAZEL.

	**Effort-reward imbalance**	
**Job strain**	No	Yes
No	7160 (75.2)	1060 (11.1)
Yes	878 (9.2)	426 (4.5)

Table S4g. Numbers (%) of participants with effort-reward imbalance and job strain in HNR.

	**Effort-reward imbalance**	
**Job strain**	No	Yes
No	1457 (82.2)	101 (5.7)
Yes	179 (10.1)	36 (2.0)

Table S4h. Numbers (%) of participants with effort-reward imbalance and job strain in IPAW.

	**Effort-reward imbalance**	
**Job strain**	No	Yes
No	884 (53.3)	411 (24.8)
Yes	160 (9.6)	205 (12.3)

Table S4i. Numbers (%) of participants with effort-reward imbalance and job strain in PUMA.

	**Effort-reward imbalance**	
**Job strain**	No	Yes
No	1412 (76.9)	143 (7.8)
Yes	190 (10.3)	91 (5.0)

Table S4j Numbers (%) of participants with effort-reward imbalance and job strain in Whitehall II.

	**Effort-reward imbalance**	
**Job strain**	No	Yes
No	4785 (47.2)	3929 (38.8)
Yes	229 (2.3)	1187 (11.7)

Table S4k. Numbers (%) of participants with effort-reward imbalance and job strain in WOLF-F.

	**Effort-reward imbalance**	
**Job strain**	No	Yes
No	2687 (74.4)	483 (13.4)
Yes	244 (6.8)	197 (5.5)

Table S4l. Numbers (%) of participants with effort-reward imbalance and job strain in WOLF-S.

	**Effort-reward imbalance**	
**Job strain**	No	Yes
No	3977 (72.3)	637 (11.6)
Yes	620 (11.3)	265 (4.8)
